# Electrochemical Synthesis of Zinc Oxide Nanostructures on Flexible Substrate and Application as an Electrochemical Immunoglobulin-G Immunosensor

**DOI:** 10.3390/ma15030713

**Published:** 2022-01-18

**Authors:** Bernardo Patella, Nadia Moukri, Gaia Regalbuto, Chiara Cipollina, Elisabetta Pace, Serena Di Vincenzo, Giuseppe Aiello, Alan O’Riordan, Rosalinda Inguanta

**Affiliations:** 1 Dipartimento di Ingegneria, Università degli Studi di Palermo, 90128 Palermo, Italy; bernardo.patella@unipa.it (B.P.); nadia.moukri@unipa.it (N.M.); gaia.regalbuto@community.unipa.it (G.R.); giuseppe.aiello02@unipa.it (G.A.); 2 Fondazione Ri.MED, 90133 Palermo, Italy; ccipollina@fondazionerimed.com; 3 Istituto per la Ricerca e l’Innovazione Biomedica (IRIB)-Consiglio Nazionale delle Ricerche, 90153 Palermo, Italy; elisabetta.pace@cnr.it (E.P.); serena.divincenzo@irib.cnr.it (S.D.V.); 4 Tyndall National Institute, University College Cork, T12 R5CP Cork, Ireland; alan.oriordan@tyndall.ie

**Keywords:** zinc oxide, nanorod, immunosensors, electrodeposition, immunoglobulin-G, nanostructured materials, electrochemical sensors

## Abstract

Immunoglobulin G (IgG), a type of antibody, represents approximately 75% of serum antibodies in humans, and is the most common type of antibody found in blood circulation. Consequently, the development of simple, fast and reliable systems for IgG detection, which can be achieved using electrochemical sandwich-type immunosensors, is of considerable interest. In this study we have developed an immunosensor for human (H)-IgG using an inexpensive and very simple fabrication method based on ZnO nanorods (NRs) obtained through the electrodeposition of ZnO. The ZnO NRs were treated by electrodepositing a layer of reduced graphene oxide (rGO) to ensure an easy immobilization of the antibodies. On Indium Tin Oxide supported on Polyethylene Terephthalate/ZnO NRs/rGO substrate, the sandwich configuration of the immunosensor was built through different incubation steps, which were all optimized. The immunosensor is electrochemically active thanks to the presence of gold nanoparticles tagging the secondary antibody. The immunosensor was used to measure the current density of the hydrogen development reaction which is indirectly linked to the concentration of H-IgG. In this way the calibration curve was constructed obtaining a logarithmic linear range of 10–1000 ng/mL with a detection limit of few ng/mL and good sensitivity.

## 1. Introduction

Zinc Oxide (ZnO) is a multifunctional material [1,2] with semiconducting properties (p-type, band gap of 3.37 eV) [3,4] that is used in many advantageous applications from biomedical field, to electrical and electronics industry and energy conversion [5,6,7]. It is a cheap and biocompatible material widely used for production of paints, rubber, plastic and pharmaceuticals [8,9]. Zinc oxide is also used in piezoelectric transducers, light emitting devices, photo-sensors and chemical/electrochemical sensors [10,11,12,13,14]. In addition, due to photocatalytic and self-cleaning properties of ZnO, application in purification of wastewater by photocatalytic reaction was also proposed [15].

Commercial methods for production of ZnO thin films are gas phase based techniques such as sputtering and metal organic vapor deposition [16,17,18,19,20,21,22]. These methods are highly expensive for both instrumentations and operational conditions and they also require specialized personnel. On the contrary, liquid phase techniques, such as electrodeposition [23], sol-gel [24], microwave synthesis [25] and hydrothermal methods [26], are very simple to carry out and thus can efficiently reduce the overall cost of ZnO production. Electrodeposition, the most used method to deposit ZnO, is based on electro-generation of a base [27,28,29,30] that leads to the deposition of ZnO starting from a solution containing Zn^2+^ and nitrate ions. In particular, the electro-reduction of nitrate to nitrite leads to the generation of OH^−^ at electrode/electrolyte interface with a consequent increase of local pH [31]. This increase in pH allows the precipitation of ZnO on the electrode surface. This method, showed by Izaki et al. [32] and Peulon et al. [33], was further investigated in many studies [23,34], demonstrating that, by tuning the electrodeposition parameters, it is possible to obtain ZnO with different morphologies such as thin film, self-assembled hexagonal nanorods (NRs), nanofibers, nanoparticles, nanorings and nanowires [35,36,37,38,39,40,41,42,43,44,45,46]. The ability to obtain ZnO in nanostructured form is extremely interesting because nanostructured morphology allows electrodes with higher surface area and thus higher reactivity to be fabricated [47]. These properties are of great importance in many applications, especially for batteries [48,49], electrolyzes [50,51,52], solar cells [53,54], magnetic devices [55,56] and sensors [57,58,59]. In this work we explored the possibility of using electrochemically obtained ZnO nanorods (ZnO-NRs) as a basis for the fabrication of immunosensors for human immunoglobulin G (H-IgG). The choice of H-IgG is based on the fact that it is the most common type of antibody found in blood circulation, representing approximately 75% of serum antibodies in humans, and it is therefore a good model analyte for proof-of-concept development of innovative biosensors that may also be used in real world applications. Recently, for example, the electrochemical-based serological detection of bovine IgG was used to determine passive transfer of antibodies from colostrum to calves [60].

To date few papers have reported data on ZnO-based immunosensors. Legionella pneumophila [61], cortisol [62], ovarian cancer antigen CA-125/MUC126 [63], human salivar alpha-amylase [64], and urine albumin [65] have been detected using ZnO-based immunosensors. In the case of H-IgG detection, ZnO was used to build field-effect transistor [66] and electrochemical sensors [67]. In all these reports, however, ZnO was obtained using complicated and expensive methods while herein we propose a simple and fast method based on electrochemical deposition. In particular, in the present work, we first optimized the electrodeposition process of ZnO-NRs, studying the effect of many parameters such as temperature, pH of deposition bath, deposition time, applied potential, supporting electrolyte, zinc nitrate concentration and dissolved oxygen in order to obtain reproducible deposition of ZnO-NRs. Finally, the ZnO-NRs electrode was modified with a thin layer of reduced graphene oxide (rGO) and used as immunosensor for H-IgG.

The development of new kinds of analytical devices able to carry out fast, accurate, and real time analysis in situ is of great importance in many different fields, from health [68,69,70,71], to sport [72,73,74], homecare and environment among others [58,73,75,76,77,78,79]. Electrochemical immunosensors are perfect candidates to achieve this as they are cheap and portable. Indeed, electrochemistry, especially when used in combination with nano-sized electrodes, has excellent features, because it can provide rapid, simple, low-cost, and sometimes on-site detection [80,81]. Furthermore, electrochemical immunosensors are gaining importance because they can be used to replace old and lab-based optical techniques. These sensors allow the determination of proteins with an easier protocol, with smaller and cheaper instrumentation [82,83]. In order to detect proteins, a sandwich configuration may be constructed on the surface of the electrode [84]. The sandwich configuration consists of (a) a primary antibody attached on the electrode surface, (b) the antigen to be detected (analyte) that is selectively bound by the primary antibody and (c) a secondary labelled antibody. This secondary antibody can be labelled with several probes such as gold, platinum, silver nanoparticles or enzymes [85,86,87,88,89]. These probes give an electrical signal that is proportional to the secondary antibody concentration and therefore to the analyte concentration hence it can be used as sensor output [90]. Considering the high surface area of ZnO-NRs, this electrode can be used as ideal substrate for a sandwich-type assay, enabling high density of attached antibodies, and so high sensitivity. Furthermore, immunosensors based on ZnO display enhanced properties [91,92,93] because the high isoelectric point (IEP) of ZnO that is > pH 8 and thus it is negatively charged. On the other side, at physiological pH, many biomolecules have a IEP below physiological pH, and thus are positively charged; thus those biomolecules can be easily immobilized on the ZnO surface by simple electrostatic interactions [94]. In addition, in this work, the surface of ZnO-NRs has been modified with rGO in order to increase the capability of the electrode to bind with the primary antibody. This material has different functional groups such as -OH, -COOH, that through different chemical reactions can be converted in amino groups [95,96]. These groups easily react with proteins, increasing the deposition yield of the primary antibody. To tag the secondary antibody, gold nanoparticles (Au-NPs) were used for their capability to catalyze the Hydrogen Evolution Reaction (HER) thus allowing the detection using chronoamperometry in an acidic media. The applicability of the ZnO-NRs/rGO based electrode as immunosensor for H-IgG was demonstrated by chronoamperometry measuring the current of HER using a phosphate buffer solution.

## 2. Materials and Methods

### 2.1. Fabrication of ZnO-NRs/rGO

A conductive film of Indium Tin Oxide supported on Polyethylene Terephthalate (ITO-PET) was used as substrate to manufacture the immunosensors. In particular, flexible sheets of ITO-PET with 60 Ω cm^−2^ resistance were used (Sigma Aldrich, Darmstadt, Germany). This substrate was selected because it is cheap and has good chemical and mechanical properties. Before use, the ITO-PET sheet was cut into small pieces of 1.5 × 7 cm^2^. Each piece was treated to remove any impurities present on the surface in order to obtain a uniform deposition of zinc oxide nanorods. The pre-treatment of the ITO-PET consists of two successive washes of 15 min, first in acetone and then in isopropanol, both carried out in an ultrasonic bath (Bandelin, Sonorex Super, Berlin, Germany). Finally, the electrode was rinsed with distilled water and dried with a flow of nitrogen. Subsequently, the working surface (about 4 cm^2^) for the electrodeposition of the ZnO was defined by means of an insulating lacquer. The electrochemical depositions were carried out using a PARSTAT mod 2273 potentiostat/galvanostatic (Princeton Applied Research, Oak Ridge, TN, USA).

The electrodeposition of ZnO-NRs was carried out potentiostatically in an inert atmosphere under a continuous flow of nitrogen (1 cm^3^ min^−1^) using a 3-electrodes cell, with a Pt mesh used as a counter-electrode and a silver-silver chloride (Ag/AgCl) reference electrode. During the deposition the temperature was kept constant at 60 °C. An aqueous solution of ZnCl_2_ (zinc chloride, 99%, Alfa Aesar, Germany) and NaNO_3_ (sodium nitrate, 99%, Alfa Aesar), was used as electrolyte [4]. The pH was adjusted to a value of about 5.5, by adding suitable quantities of aqueous solution of hydrochloric acid (HCl, 36%, Alfa Aesar). The electrodeposition process was optimized with the aim to obtain a uniform distribution of ZnO-NRs, by investigating the effect of various parameters. In particular, the need to operate in an inert atmosphere, the effect of the potential (from −0.8 to −1.0 V vs. Ag/AgCl), temperature (60 and 80 °C), pH (from 4 to 6.8), concentration of ZnCl_2_ (5, 10, 15 mM), type of support electrolyte (potassium chloride (KCl) and sodium chloride (NaCl), 98%, Alfa Aesar) and the concentration of NaNO_3_ (10, 50, 100 mM) were investigated. For each investigated parameter, three different electrodes were obtained and tested.

Subsequently, the rGO was deposited on the substrate consisting of the ZnO nanorods, by means of electrochemical reduction. The deposition of rGO took place potentiostatically by applying a cathodic potential of −0.8 V (Ag/AgCl) for 300 s using a 4 mg/mL GO solution (Graphenea, Cambridge, MA, USA), diluted in phosphate buffer solution (PBS, pH 7.4, (Sigma Aldrich)) up to a concentration of 0.5 mg/mL.

Samples were characterized by means of scanning electron microscopy (SEM, FEG-ESEM, FEI QUANTA 200, OR, USA), energy dispersive spectroscopy (EDS, EDAX, Ametek, PA, USA), X-ray diffraction (XRD, RIGA, D-MAX 25,600 HK, Tokyo, Japan) and Raman spectroscopy (Renishaw, inVia Raman Microscope, UK). Characterization methods have been detailed in our previous reports [97,98,99,100,101,102].

### 2.2. Fabrication of Immunosensors

The optimized ITO-PET/ZnO NRs/rGO based electrodes were used to the fabrication of immunosensors with a sandwich configuration. Firstly, Au-NPs, necessary to tag the secondary antibodies, were synthesized following the Turkevich method [103]. Briefly, a volume of 50 mL of 0.25 mM AuHCl_4_ (Hydrogen tetrachloroaurate, 99.99%, Alfa Aesar) was heated until it started to boil under vigorous stirring. Then, 1.25 mL of 1% sodium citrate (Na_3_C_6_H_5_O_7_, 99%, Alfa Aesar) was quickly added to the boiling solution, and a gradual color change, from purple to red, was observed. The heater was switched off once an appropriate red color was observed. Finally, the solution was cooled down and stored at 4 °C. The Au-NPs were then conjugated with antibodies against H-IgG. Prior to this step, NPs suspension was centrifugated and the NPs were suspended in borate buffer. In order to conjugate anti-H-IgG antibodies with AuNPs, 72 µL of borate buffer (pH 9.2, Sigma Aldrich) and 67 µL of anti-H-IgG (250 µg/mL, Sigma Aldrich) were added per mL of NPs solution. The prepared solution was mixed for 20 min at 4 °C and 650 rpm. Subsequently, 67 µL of Bovine Serum Albumin (BSA, 98% Sigma Aldrich) was added in order to block the unreacted NPs and avoid agglomeration and precipitation. After addition of BSA, the solution was stirred for 20 min and 4 °C at 650 rpm. Finally, excess antibodies and BSA were removed by centrifugation at 13,000 rpm, for 30 min and 4 °C. The Au-NPs conjugated with anti-H-IgG antibodies were then suspended in PBS (75 µL of PBS and 350 µL of Au-NPs).

**Figure 1:**
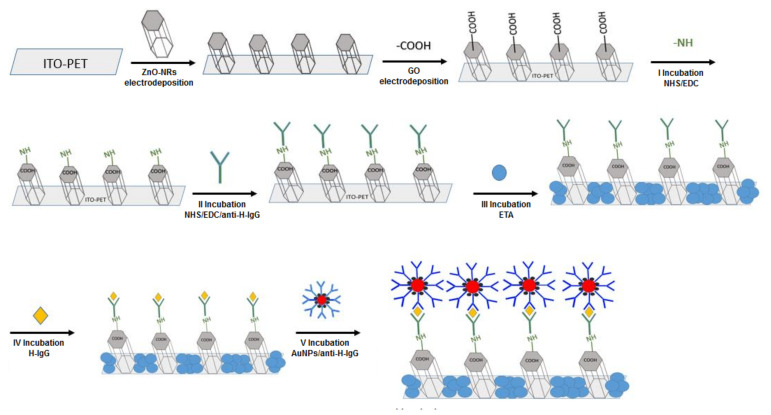
Scheme of the fabrication of immunosensor with a sandwich configuration based on ZnO nanorods.

Different incubation steps were necessary to obtain immunosensors with a sandwich configuration on ZnO-rGO based electrodes (Figure 1). Incubations were performed using home-made cells with a small volume of ~50 µL. The cells were developed using a 3D printer (Zortrax mod. M200). The exposed geometric area of the electrode (ITO-PET/ZnO NRs/rGO) was ~0.07 cm^2^.

The first incubation step aimed at increasing the affinity between the antibody and the electrode, through the functionalization of the electrode surface with amino groups in order to facilitate the immobilization of the H-IgG. For this step, the sensor was immersed in a solution consisting of 3 mM N-hydroxysuccinimide (NHS, C_4_H_5_NO_3_, 98%, Sigma Aldrich) and 15 of mM 1-ethyl-3-(3-dimethylaminopropyl)-carbodiimide hydrochloride (EDC, C_8_H_17_N_3_-HCl, 98%, Sigma Aldrich) in PBS for one hour. Then, the antibody immobilization step was carried out by immersing the sensor in presence of 3 mM NHS, 15 mM EDC and 0.2 mg/mL of antibodies (second step) overnight at room temperature. Then, a blocking step to avoid non-specific interactions was undertaken by immersing in 0.5 M ethanolamine (ETA, NH_2_CH_2_CH_2_OH, 99%, Sigma Aldrich) solution for 1 h at room temperature (third step). This step is very important as non-specific signals can occur from the presence of Au-NPs on the electrode surface deposited during the incubation of the secondary antibody tagged with Au-NPs but in the absence of the target H-IgG. The fourth incubation step was conducted with different concentrations of H-IgG antigen (from 1 to 1000 ng/mL) for 1 h at room temperature, to allow antigens to fully bind to the primary antibodies. Finally, in the last step the primary antibody-antigen complex was incubated with the Au-NP labelled secondary antibody for 1 h at room temperature. Following each incubation step, the substrates were washed three time with pure PBS, in order to eliminate excess reagents.

A chronoamperometry approach, performed by imposing a constant potential of −0.9 vs. saturated calomel electrode (SCE), was employed for signal detection and readout. The current was recorded until a stable signal was reached (variation less than 0.1 µA/sec).

For each experiment described in this work, a new electrode with the same features was used, making three or five replicate tests.

## 3. Results and Discussion

The electrodeposition process of ZnO, introduced by Izaki et al. [32], has several advantages such as low cost, environmental friendly and easy scalability. The deposition of ZnO is an electrochemically induced precipitation process [47,104] and occurs from an aqueous solution of zinc salts (ZnCl_2_ or Zn(NO_3_)_2_). Depending on the deposition solution, dissolved oxygen (reaction 1) or nitrate ions (reaction 2) or both, react producing OH^−^ close to the electrode/electrolyte interface. The electro-generation of base leads to an increase of interfacial pH allowing the precipitation of zinc hydroxide (reaction 3) on the surface of working electrode. Then, zinc hydroxide easily dehydrates to zinc oxide (at temperature generally >35 °C) following (reaction 4). For electrodeposition baths containing ZnCl_2_ at temperature above 50 °C, ZnCl^+^ is the predominant species present in solution, thus the deposition of ZnO can be also attributed to (reaction 5).

(1) $$O_{2}+2H_{2}O+4e^{-}=4OH^{-}$$

(2) $$NO_{3}^{-}+H_{2}O+2e^{-}=NO_{2}^{-}+2OH^{-}$$

(3) $$Zn^{2+}+2OH^{-}=Zn{\left(OH\right)}_{2}$$

(4) $$Zn{\left(OH\right)}_{2}=ZnO+H_{2}O$$

(5) $$Zn{\mathrm{Cl}}^{+}+2OH^{-}=ZnO+H_{2}O+Cl^{-}$$

From these reaction mechanisms, as demonstrated in [4,46], it is clear that the deposition of ZnO depends upon many parameters such as the initial pH of deposition solution, temperature, zinc salt concentration, presence of dissolved oxygen. Besides, as shown in [105], a crucial role in the morphology of electrodeposited ZnO is also played by the conductive substrate used as working electrode. Consequently, to optimize the deposition process it was necessary to fine tune all these parameters. Considering that chloride-based baths are the most suitable for an efficient growth of ZnO nanorods [46,106] on ITO substrates [105], in this work zinc chloride was selected as a zinc source while nitrate ions were added as sodium nitrate to increase the rate of base generation.

**Figure 2:**
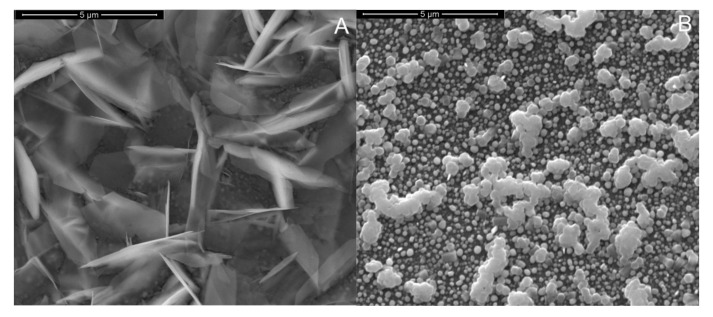
SEM images of samples obtained using a solution of 10 mM ZnCl_2_ + 10 mM NaNO_3_ at 60 °C for 180 min at applied potential −1 V (**A**) with saturated dissolved oxygen and (**B**) following N_2_ purge degassing.

The effect of dissolved oxygen in deposition solution was explored by performing different experiments in a solution of ZnCl_2_ and NaNO_3_ (both 10 mM) at 60 °C, −1 V for 180 min in aerated and de-aerated condition (under a continuous N_2_ flux,) using Ag/AgCl as reference. In aerated condition, randomly orientated nanosheets of ZnO were observed using scanning electron microscopy, see Figure 2A in agreement with the data reported in [107]. A different morphology was observed using de-aerated solutions. The SEM image in Figure 2B shows the presence of some rods with hexagonal shape characteristic of ZnO with wurtzite-type structure [46]. Considering that for sensing applications the nanorod is the preferred morphology, due to its high surface area, all further growth experiments were carried in de-aerated solution. Furthermore, the inert atmosphere should increase the reproducibility of the process, since it avoids the fluctuation of oxygen concentration that is another important parameter influencing the ZnO morphology [46].

As reported in [46], the presence of NaCl or KCl, acting as supporting electrolytes, is a key factor to control the density of nanorods array. To study this aspect, depositions were performed in de-aerated solutions containing (i) 10 mM of ZnCl_2_, 10 mM NaCl and 10 mM NaNO_3_ and (ii) 10 mM of ZnCl_2_ and 10 mM NaNO_3_ (absence of NaCl). The electrodeposition was carried out for 60 min at −1 V vs. Ag/AgCl and 60 °C. The deposition time was reduced to 60 min, because results shown in Figure 2B indicate that a deposition time as long as 180 min favors the lateral growth of the nanorods. This arises from the coalescence phenomena, i.e., where neighboring nanostructures coalesce to form microrods, clearly visible in Figure 2B, which would eventually lead to a decrease of the surface area. Similar results were obtained using KCl as supporting electrolyte [108].

**Figure 3:**
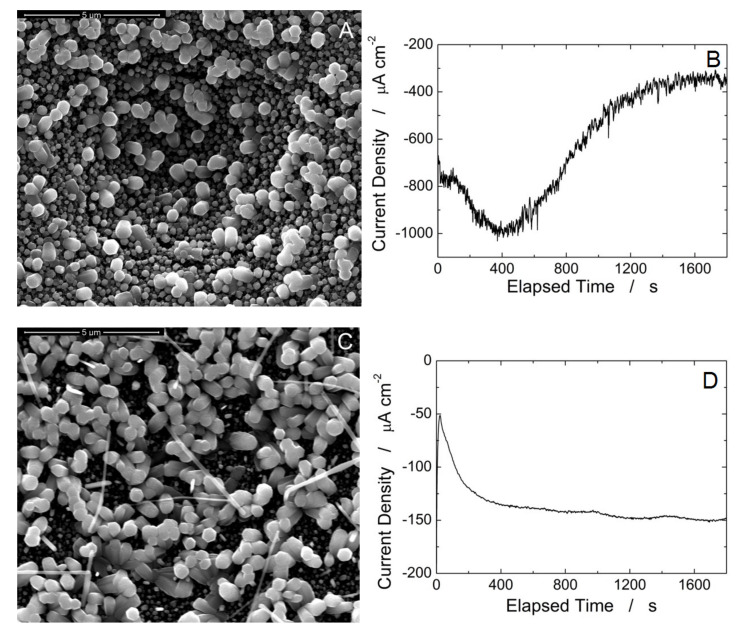
SEM image of samples obtained at 60 °C for 30 min at applied potential −1 V under N_2_ atmosphere (**A**) with 10 mM ZnCl_2_ + 10 mM NaCl + 10 mM NaNO_3_ and (**B**) corresponding deposition curve, (**C**) with 10 mM ZnCl_2_ + 20 mM NaNO_3_ and (**D**) corresponding deposition curve.

Figure 3 shows the morphology of the electrode surfaces, and the corresponding chronoamperometry curves, for deposition carried out with (Figure 3A,B) and without NaCl (Figure 3C,D). Figure 3B is a typical growth curve of a fast deposition process, with a rapid increase in the current density and a subsequent decrease due to the consumption of the Zn precursor concentration in solution. In the presence of NaCl, the deposition current (Figure 3B) is higher, due to the increase of solution conductivity. Without the NaCl (Figure 3D) a reduced current was observed and the shape of current density curves is also different. The curve in Figure 3D is typical of a slow process, in which after the initial transient, the current density remains approximately constant over time. A similar trend was reported in the literature in the case of electrodepositions carried out at different temperatures [46]. In particular, the curve of Figure 3B is typical of a high temperature process and therefore kinetically fast (>40 °C), while Figure 3D is typical of processes conducted at low temperature and therefore slow (<35 °C). This obviously influences the morphology of the ZnO deposit. The high current density in the presence of NaCl leads to a high nucleation of ZnO sites, as shown in Figure 3A. Under these conditions there is a high density of nanorods (nanoparticles adhering together) with extremely uneven lengths, typical of fast growth processes. Therefore, the increase in current using NaCl as support electrolyte leads to the formation of ZnO with an irregular morphology which could cause reproducibility problems of the sensor performances. For the low current densities, Figure 3C, the density of nucleation sites is lower. In this way, fewer nanorods were formed, but with much more uniform lengths. For this reason, we have chosen the solution without NaCl support as the most suitable for the subsequent tests.

**Figure 4:**
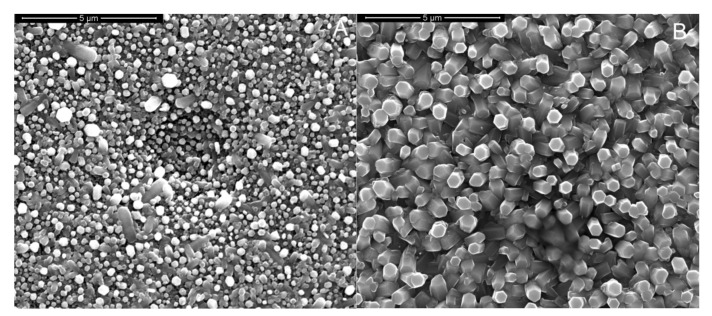
SEM images of the samples obtained in a de-aerated solution of 10 mM ZnCl_2_ 10 mM NaNO_3_ at −1V: (**A**) 80 °C, 60 min, untreated pH 6.5–6.8, (**B**) 60 °C, 60 min, pH 4.5.

To further understand the mechanism of ZnO deposition, the effect of temperature and pH of the deposition bath were also studied. The temperature was changed from 60 to 80 °C with a de-aerated solution of 10 mM ZnCl_2_ and 10 mM NaNO_3_ whilst applying −1 V vs. Ag/AgCl for 60 min. Figure 4A shows the SEM image of the electrode deposited at 80 °C. In comparison to the sample obtained at 60 °C (Figure 3C) where well defined ZnO NRs were clearly visible, at 80 °C the high deposition rate led to the formation of high density nanorods really small, very close to each other to almost form a continuous film. Of note, at 80 °C the growth curve is similar to that of Figure 3B, but with a value of current density twice as high and a shorter transient. Thus, to obtain an electrode with high surface area, main goal of this work, the optimal deposition temperature that was selected for further work is 60 °C.

All depositions described above were performed without modifying the pH of as-prepared solution that ranged in the interval 6.5–6.8 pH, together with temperature, is one of the fundamental parameters that controls the deposition process of ZnO. In fact, the solubility equilibrium of the zinc oxide and the hydroxide depends on the pH and the temperature. The speed with which the precipitation pH is reached at the interface (in our conditions around 6.5–6.8 [46]) has a notable influence on the morphology of the ZnO deposit, as it affects the nucleation rate. In particular, it has been shown that the diameter and the growth density of ZnO NRs are mainly defined during the initial nucleation phase. For low nucleation rates, large and scattered ZnO grains have been obtained which carry a uniform and regular series of NRs [109].

To reduce the nucleation rate, it may be useful to use a low acid solution. To verify this, a de-aerated solution of 10 mM ZnCl_2_ and 10 mM NaNO_3_ at pH 4.5 was used. The solution was acidified with HCl and kept under vigorous stirring for 12 h. Interestingly, when the deposition was carried out at pH 4.5, the NRs morphology was more homogenous, Figure 4B, with the formation of an array of ZnO NRs with a well-defined hexagonal structure. The decrease of the initial pH of the solution, maintaining all other parameters equal, allowed a better control of the nucleation stage. In this way, at the start of the deposition process, a few nucleation sites were formed on the surface of the electrode which then grow in an orderly and uniform way. This same effect has been obtained by adding NH_4_NO_3_ into the solution that acts as a pH-buffering agent. Considering these results, the following experiments were carried out using a solution at pH 4.5.

**Figure 5:**
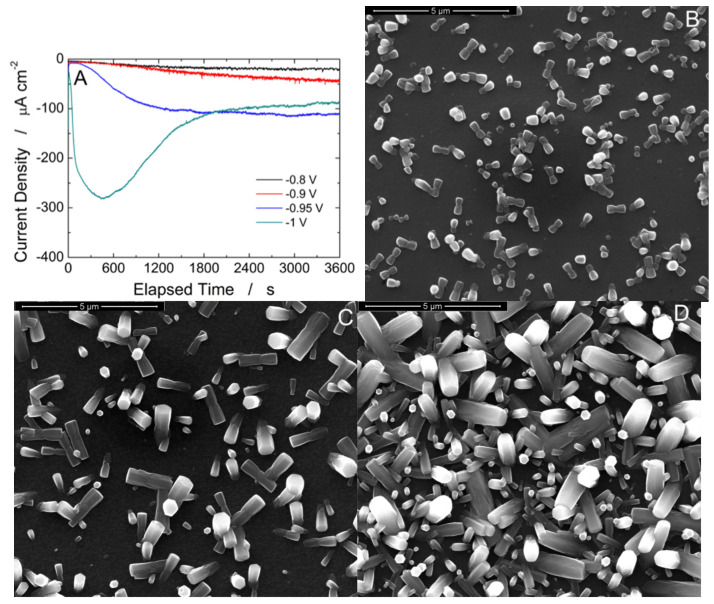
(**A**) Current density curves and SEM images (**B**) −0.8 V, (**C**) −0.9 V, and (**D**) −0.95 V vs. Ag/AgCl) of electrodes obtained using a de-aerated solution of 10 mM ZnCl_2_ and 10 mM NaNO_3_ at pH 4.5 for 60 min at 60 °C.

**Figure 6:**
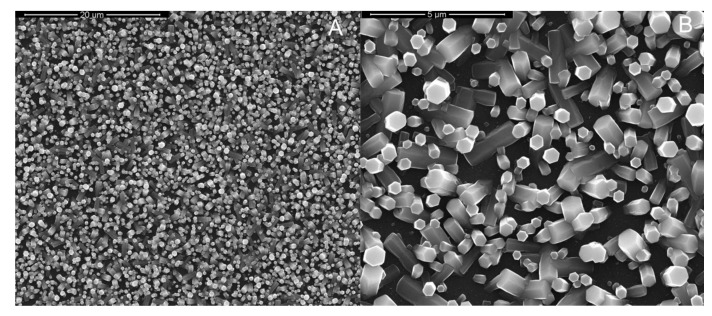

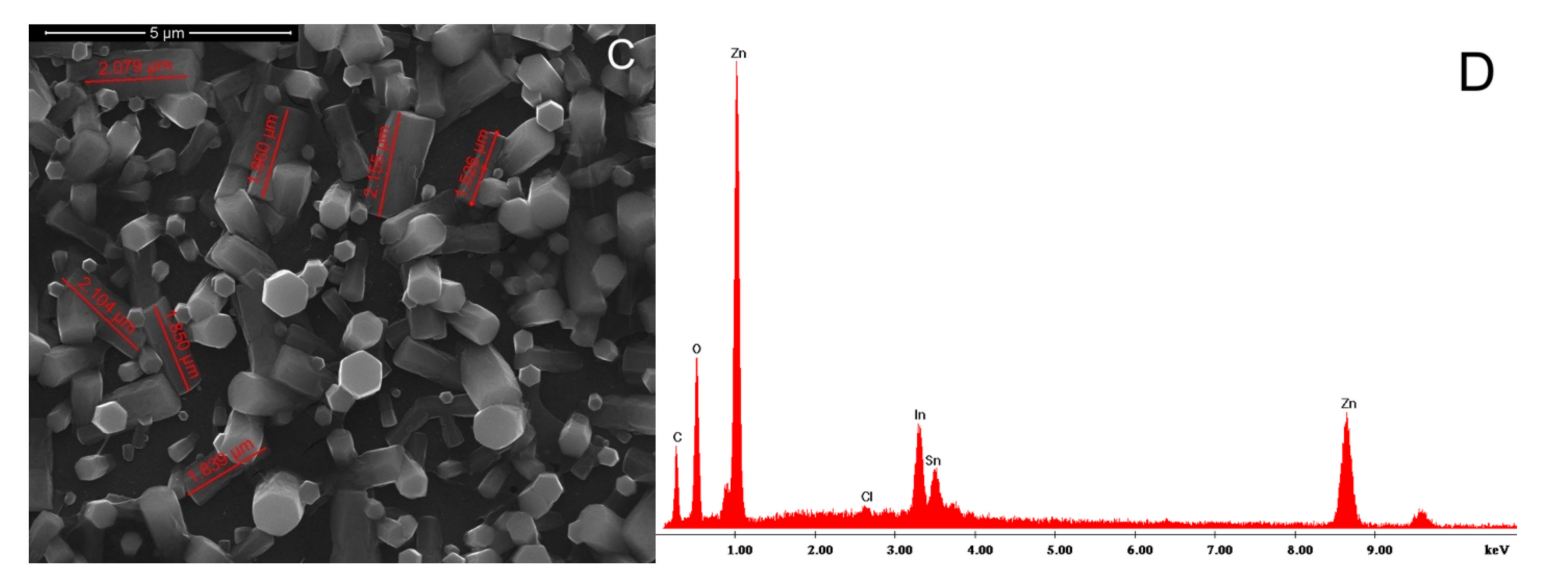
SEM images (**A**–**C**) of ZnO nanorods and (**D**) EDS spectrum of the optimized electrode. The electrode was obtained at −0.95 V vs. Ag/AgCl for 60 min in a de-aerated solution of 10 mM ZnCl2 and 10 mM NaNO_3_ at pH 4.5.

The effect of applied potential was studied in the range from −0.8 V to −1.0 V vs. Ag/AgCl and is presented in Figure 5. As expected, the increase of the applied cathodic potential, resulted in higher current densities. For applied potential lower than −0.95 V, the current density increased over time and tended to reach a steady state, Figure 5A. For depositions carried out at −1 V a different shape was observed. As above discussed, the different shape in current density curves is due to the different deposition rate, low at low applied cathodic potential and vice versa. Obviously, this is reflected in the morphology of the zinc oxide and confirms our previous discussions. Similar results and conclusions were also gained by Dalchiele et al. [104]. For applied cathodic potentials lower than −0.8 V, the deposition rate is low, consequently the nucleation rate is low. Under these conditions few ZnO NRs (Figure 5B) sparse on the electrode surface are formed. Increasing the applied cathodic potential, −0.9 V, increases the density of the NRs and also their diameter, as clearly visible in Figure 5C. As reported in [110], the morphology of Figure 6B, consisting of disordered ZnO NRs without a preferential texture, is obtained using substrate without seed layer. In the case of depositions performed at −0.95 V, the entire electrode surface is covered by ZnO NRs with a consequently very high surface area, Figure 5D. As reported in [111], this morphology is ideal to immobilize molecules on the surface of NRs because it allows the lateral surface to be fully exploited without problems of solution permeation and of reciprocal steric hindrance. A further increase in the applied potential, −1 V, favors the lateral growth of NRs, with a reduction of the interspatial distances between nanorods (Figure 4B) leading to the formation of a very compact array and thus a lower surface area. In addition, this type of morphology is characterized by a low wettability [112] and should be avoided. Thus, to obtain a good compromise between the density of the NRs and a high active surface area an applied cathodic potential of −0.95 V vs. Ag/AgCl was selected.

The effect of NaNO_3_ concentration was studied in the range from 10 to 100 mM. In this case no noticeable difference in morphology of the electrode can be observed. In fact, only a small increase in the size of the NRs was observed. This is also confirmed by the growth curves that, apart from small differences in the current density values (slightly higher as the concentration of NaNO_3_ increases) were practically identical.

Thus, the optimal conditions to obtain an array of ZnO NRs using a potentiostatic deposition were: −0.95 V vs. Ag/AgCl for 60 min in a de-aerated solution of 10 mM ZnCl_2_ and 10 mM NaNO_3_ at pH 4.5. The optimized electrodes were further characterized by SEM, EDS and Raman. The SEM images in Figure 6A–C show the morphology at different magnifications. The entire surface of the electrode, Figure 6A, was covered with ZnO NRs with a mean length of about 0.8 µm. The sides of the hexagonal head of the NRs were observed to have different sizes (average size between 270 and 460 nm) and two types of morphology. In Figure 6B,C both hexagonal NRs along the entire length and NRs with dumbbell-shaped morphology are clearly observed [113]. A possible formation mechanism of dumbbell-shaped ZnO NRs was proposed by Lu et al. [114]. Figure 6D shows the EDS spectrum with the presence of C arising from the PET substrate, Sn and In from the ITO film, O from both PET substrate and ZnO and obviously the source of the Zn peak.

In order to increase the immobilization of the primary antibody, rGO was deposited on top of the ZnO NRs optimized electrodes using electrodeposition by applying a constant potential [115]. GO stock solution (4 mg/mL) was dispersed in acetate buffer solution with a final concentration of 0.5 mg/mL and the ITO-PET substrate coated with ZnO NRs was mounted in home-made cell exposing an area of 0.07 cm^2^ to operate as working electrode. A rod of platinum and SCE were used as counter and reference electrodes, respectively. A cathodic potential of −0.8 V (SCE) for 300 s was applied so that the following (reaction 6) [116] takes place at electrode/electrolyte interface leading to formation of rGO on the surface of the NRs

(6) $$GO+xH^{+}+ye^{-}\to rGO+zH_{2}O$$

**Figure 7:**
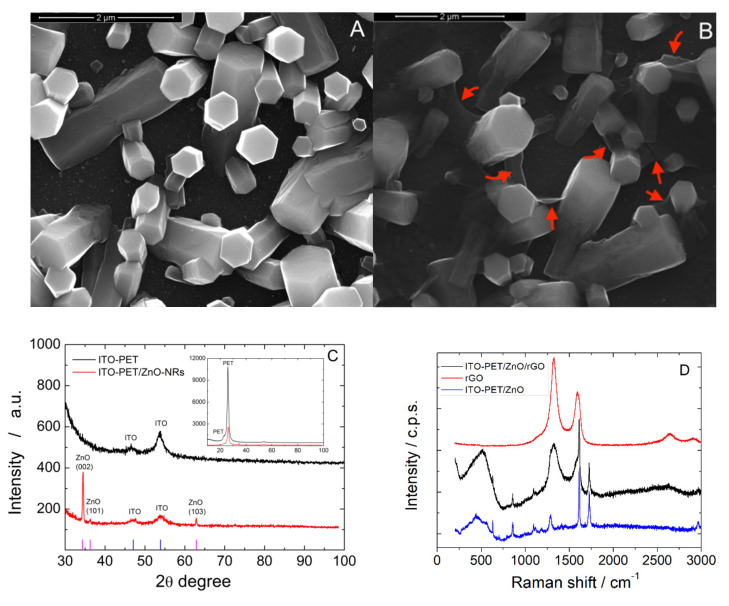
(**A**,**B**) FESEM images (**A**) before and (**B**) after rGO deposition, (**C**) XRD patterns and (**D**) Raman spectra. The ZnO electrode was obtained at −0.95 V vs. Ag/AgCl for 60 min in a de-aerated solution of 10 mM ZnCl2 and 10 mM NaNO_3_ at pH 4.5.

As it can be observed in the high magnification images presented in Figure 7, after rGO deposition, the electrode was covered with a thin film of rGO that is clearly evident in the marked areas of Figure 7B. As expected, the rGO is extremely thin and its presence does not change the morphology of the ZnO NRs [117]. To better point out the presence of rGO in Figure 7B, the high magnification SEM image of electrode before rGO deposition is also reported, Figure 7A.

The rGO-ZnO-NRs coated electrodes were characterized by XRD and Raman spectroscopy to confirm the effectively presence of a ZnO and rGO. Figure 7C shows the XRD patterns of the electrode. The high intensity diffraction peaks at 34.3, 36.2 and 62.9° match with the ZnO hexagonal wurtzite structure (ICDD card 36-1451) [111,114]. As reported in [104], the high intensity of (002) diffraction plane is attributed to the presence of well-shaped c-axis oriented hexagonal columns. The presence of rGO cannot be verified by XRD, because its pattern is characterized by the presence of only a broad band located at about 26°, thus overlapped onto the main peak of the PET substrate. To demonstrate rGO deposition Raman spectroscopy was performed. In Figure 7D, the spectrum of rGO only is reported for comparison. In the Raman spectrum of rGO two main peaks at about 1350 and 1600 cm^−1^, corresponding to the D and G band [118], respectively, can be observed. These peaks were found also in the rGO-ZnO-NRs electrode confirming the deposition of rGO. Particularly, the peak ratio between D and G bands in rGO-ZnO-NRs electrode is of about 1.5 showing the efficient reduction of GO. The broad band at 500 cm^−1^ is characteristic of ZnO, and is generated by the overlapping of the vibrational modes at 310, 440 and 580 cm^−1^ [117]. The other Raman modes coming from ITO/PET substrate [4].

The deposition of rGO, completes the electrode substrate fabrication and its characteristics (high surface area, biocompatibility, conductivity) constitutes an ideal conductive support for the realization of biosensors using a sandwich configuration. To confirm this, biosensors for the H-IgG model protein were developed using the optimized ITO-PET/ZnO NRs/rGO electrodes following the proposed procedure [67,86].

**Figure 8:**
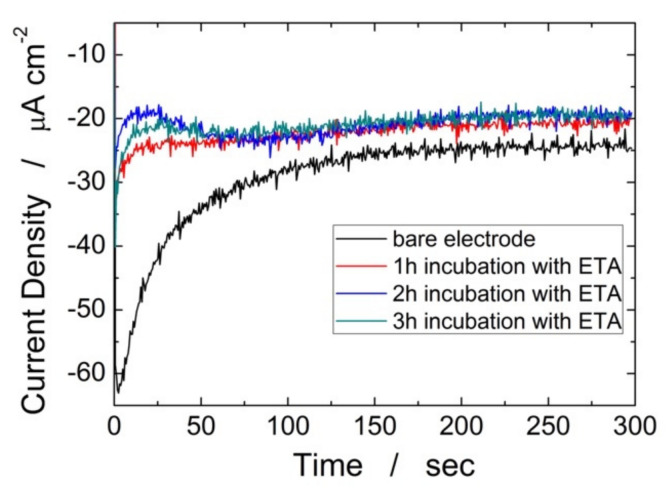
Chronoamperometric curves before and after incubation of ITO-PET/ZnO NRs/rGO with ETA. The ZnO electrode was obtained at −0.95 V vs. Ag/AgCl for 60 min in a de-aerated solution of 10 mM ZnCl2 and 10 mM NaNO_3_ at pH 4.5.

The method employed to obtain the immunosensor and to detect H-IgG consists of 5 different steps. The first incubation was carried out using a solution of 3 mM NHS and 15 mM EDC diluted in PBS for 60 min at room temperature. This incubation is a key step because it modifies the electrode surface with amino groups that can easily react with the primary antibodies. Then, primary antibody solution was incubated on the electrode surface overnight at 30 °C using a solution of 3 mM NHS, 15 mM EDC and 0.2 mg/mL of anti-H-IgG. In the subsequent step the modified electrode was treated with a solution of 0.5 M ETA for different times at room temperature. This step is fundamental for the correct functioning of the immunosensor because it allows to block all areas of the electrode not covered by the primary antibody. In this way, during the detection phase, the current signal will arise only from the sandwiches because they will be the only electrochemically active parts. The efficiency of the ETA treatment was evaluated by measuring the hydrogen evolution current density generated on the electrode surface at constant potential of −0.9 V vs. SCE in 0.1 M HCl. The effect of ETA is to block the areas that remained electrochemically active as they are not bound to the primary antibody. Consequently, a lower current should be recorded after treatment with ETA. Figure 8 shows the effect of blocking time on the chronoamperometry curves. Prior to ETA treatment, a current density of approximately −32 µA cm^−2^ was measured. After the ETA treatment for 1 h the current density decreases down to −24 µA cm^−2^. As it can be seen in Figure 8 for longer treatments, 2 and 3 h, the current density remained practically the same as the 1 h treatment. Consequently, 1 h incubation with 0.5 M ETA is sufficient to block the electrode surface. After blocking, electrodes were incubated with different amounts of target H-IgG (ranging from 1 to 1000 ng/mL) diluted in PBS for 1 h at room temperature to define a calibration plot. Finally, the electrodes were incubated (1 h room temperature) in a solution containing the secondary antibody, previously tagged with Au-NPs, to complete the sandwich. The greater the number of electrochemically active sandwiches, the greater the hydrogen developing current. By measuring this, (0.1 M HCl polarized at −0.9 V vs. SCE) the sensor calibration line was determined with different H-IgG concentrations.

**Figure 9:**
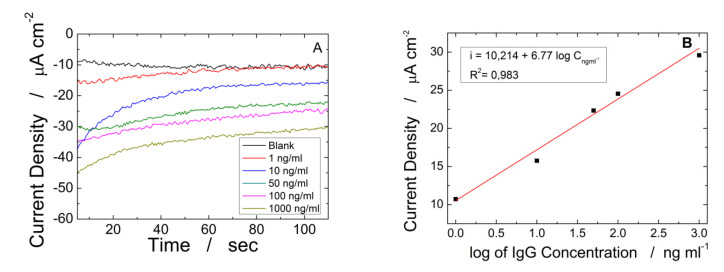
Electrochemical characterization of immunosensors fabricated on of ITO-PET/ZnO NRs/rGO substrate: (**A**) chronoamperometric curves and (**B**) respective calibration curve. The ZnO electrode was obtained at −0.95 V vs. Ag/AgCl for 60 min in a de-aerated solution of 10 mM ZnCl2 and 10 mM NaNO_3_ at pH 4.5.

In Figure 9A the effect of increasing H-IgG concentration on the chronoamperometric curves is shown. The measured current is related to the hydrogen evolution reaction that was catalyzed by the Au NPs present in the sandwich. As expected, with the increase of H-IgG, directly correlated to the number of the electrochemically active sandwiches, the current density increases with a logarithmic response. The corresponding calibration line is reported in Figure 9B. A linear range from 10 to 1000 ng/mL was observed with an estimated LOD of 1.25 ng/mL, calculated by the following equation:

(7) $$LOD=\frac{3.3*SD}{S}$$

where SD is the standard deviation of the blank and S the slope of the calibration line. From the slope of linear range, a sensitivity of 6.77 µA cm^−2^/log(ng/mL) was calculated.

Table 1 shows the presents features of the immunosensors developed in the present study (linear range, sensitivity, LOD and R^2^) and back compares them to the literature with others reporting electrochemical-based quantification of H-IgG. Considering that a logarithmic trend was found with the proposed sensor, the sensor sensitivity depends (C^−1^) on the H-IgG concentration (derivate of the fitting equation). This makes the comparison between different sensors hard to carry out. Indeed, while sensors with a linear regression have a constant sensitivity over the entire concentration range, a logarithmic trend leads to higher sensitivity for low concentration and lower sensitivity for high concentration. In order to better understand the comparison of Table 1, in the sensitivity column (only for sensors with a linear regression), the concentration at which our sensor and the considered sensor have the same sensitivity is shown. For concentration below this value, our sensor shows higher sensitivity and vice versa.

From the results of Table 1 it is clear that the developed sensors have excellent features with a really wide linear range (2 orders of magnitude) and very high sensitivity. This high sensitivity has been achieved thanks to the use of a nanostructured electrode substrate that ensures a high surface area. A further advantage of this substrate is its ease of manufacture.

## 4. Conclusions

In this work, an electrochemical immunosensor based on ZnO NRs was developed for the quantification of H-IgG. This protein is very important, and it was selected as a model analyte due to its physical, chemical and biological characteristics similar to many other proteins.

The immunosensor was developed using the ‘sandwich’ configuration in which a primary antibody is used, which binds to the target antigen, followed by a secondary antibody labeled with Au-NPs. With this configuration, the sandwich is electrochemically detectable, as the Au-NPs catalyze the hydrogen development reaction.

To make the immunosensor, an ITO-PET substrate was used on which the ZnO NRs were deposited by means of electrogeneration of a base. To obtain ZnO with nanorod morphology it was necessary to optimize the various parameters that govern the electrochemical deposition such as the deposition potential, the composition of solution and so on. The optimal conditions foresee a potentiostatic deposition at −0.95 V vs. Ag/AgCl, for 1 h, at 60 °C and in an inert atmosphere and using a bath composed of ZnCl_2_ and NaNO_3_. After the formation of the nanorods, a layer of rGO was electrochemically deposited to ensure an easy immobilization of the antibodies on the nanostructured ZnO.

The immunosensor was obtained through different incubation stages, which were all optimized in terms of time, temperature, concentration and composition of the solutions used. The immunosensor was then used to measure the current density related to the hydrogen development reaction which is indirectly related to the concentration of the H-IgG. Through these measurements, the calibration curve of the sensor was obtained in the concentration range of the H-IgG from 1 ng/mL to 1000 ng/mL. From the calibration curves it has been observed that the linear operating range of the sensor is included in the range 10–1000 ng/mL with a detection limit of 1.25 ng/mL and a sensitivity of 6.77 µA cm^−2^/log(ng/mL).

These values of both LOD and sensitivity fall within the typical values of many biosensors present in the literature, with the advantage in this case of having used an extremely cheap and easy-to-make substrate.

## Figures and Tables

**Table 1:** Comparison between electrochemical immunosensors for H-IgG. (Cys: Cysteine, PME: platinum microelectrodes, MEMS: micro electromechanical, Systems, CPE: Carbon Paste electrode, MWCNTs: Multi walled carbon nanotubes, GCE: Glassy carbon electrode, CAF: Containing aldehyde and ferrocene groups, NPs: Nanoparticles, EFP: polymer containing epoxy groups, rGO: reduced graphene oxide, PDA: polydopamine, CSPE: Carbon screen printed electrode; GO: graphene oxide, ITO: Indium tin oxide, NRs: nanorods).

	Method Linearity	Linear Range ng mL^−1^	Sensitivity µA ng^−1^ mL cm^−2^	LOD ng mL^−1^	R^2^	Ref
ZnO/chitosan	CH Linear	2.5–500	0.152 (44.5 ng^−1^ mL)	1.2	0.993	[119]
Ag@Au/Cys/Nafion/PME	CH Logarithmic	2.3–960	N.S.	10	N.S.	[120]
Au-MEMS	CH Linear	50–400	0.016 (423 ng^−1^ mL)	10	N.S.	[121]
CPE-CdFe_2_O_4_–SiO_2_	CH Logarithmic	510–30,170	7.28 ∗ C^−1^	180	0.991	[122]
Au-MWCNTs–Fe_3_O_4_	DPV Linear	30–1000	1.71 (3.95 ng^−1^ mL)	25	0.998	[123]
CPE- Bi_2_Se_3_	DPV Linear	2–300	1.86 (3.63 ng^−1^ mL)	0.8	0.992	[124]
GCE-polymer CAF	DPV Linear	0.1–20	17.63 (0.384 ng^−1^ mL)	0.07	0.997	[125]
CPE-AuNPs	DPV Linear	10–300	0.0046 (1471.7 ng^−1^ mL)	N.S.	0.983	[126]
EFP–CNTs	DPV Linear	0.1–25	0.9145 (7.4 ng^−1^ mL)	0.05	0.984	[127]
PDA-rGO-AuNPs/GCE	DPV Logarithmic	0.1–100	0.746 ∗ C^−1^	0.0075	0.99	[128]
CSPE-GO	DPV Linear	2.5–100	1.74 (3.89)	1.99	0.994	[129]
**ITO-ZnO NRs-rGO**	**CH** **Logarithmic**	**10–1000**	**6.77 ∗ C^−1^**	**1.25**	**0.982**	**This work**

## Data Availability

Data is contained within the article.
